# Profile and outcomes of hospitalized patients with COVID-19 at a tertiary institution hospital in Ghana

**DOI:** 10.4314/gmj.v54i4s.7

**Published:** 2020-12

**Authors:** Nana K Ayisi-Boateng, Michael Owusu, Phyllis Tawiah, Brenda A Ampah, Augustina A Sylverken, Osei K Wusu-Ansah, Fred S Sarfo, Richard O Phillips

**Affiliations:** 1 Department of Medicine, Kwame Nkrumah University of Science and Technology, Kumasi, Ghana; 2 University Hospital, Kwame Nkrumah University of Science and Technology, Kumasi, Ghana; 3 Department of Medical Diagnostics, Kwame Nkrumah University of Science and Technology, Kumasi, Ghana; 4 Kumasi Centre for Collaborative Research into Tropical Medicine, Kumasi, Ghana; 5 Departement of Theoretical and Applied Biology, Kwame Nkrumah University of Science and Technology, Kumasi, Ghana; 6 Komfo Anokye Teaching Hospital, Kumasi, Ghana

**Keywords:** Clinical, COVID-19, Hospitalized, Outcomes, Ghana

## Abstract

**Background:**

In high-income countries, mortality related to hospitalized patients with the Coronavirus disease 2019 (COVID-19) is approximately 4–5%. However, data on COVID-19 admissions from sub-Saharan Africa are scanty.

**Objective:**

To describe the clinical profile and determinants of outcomes of patients with confirmed COVID-19 admitted at a hospital in Ghana.

**Methods:**

A prospective study involving 25 patients with real time polymerase chain reaction confirmed COVID-19 admitted to the treatment centre of the University Hospital, Kwame Nkrumah University of Science and Technology (KNUST), Kumasi, Ghana from 1^st^ June to 27^th^ July, 2020. They were managed and followed up for outcomes. Data were analysed descriptively, and predictors of mortality assessed using a multivariate logistic regression modelling.

**Results:**

The mean age of the patients was 59.3 ± 20.6 years, and 14 (56%) were males. The main symptoms at presentation were breathlessness (68%) followed by fever (56%). The cases were categorized as mild (6), moderate (6), severe (10) and critical (3). Hypertension was the commonest comorbidity present in 72% of patients. Medications used in patient management included dexamethasone (68%), azithromycin (96%), and hydroxychloroquine (4%). Five of 25 cases died (Case fatality ratio 20%). Increasing age and high systolic blood pressure were associated with mortality.

**Conclusion:**

Case fatality in this sample of hospitalized COVID-19 patients was high. Thorough clinical assessment, severity stratification, aggressive management of underlying co-morbidities and standardized protocols incountry might improve outcomes.

**Funding:**

None declared

## Introduction

The severe acute respiratory syndrome coronavirus-2 (SARS-CoV-2) is an enveloped, single-stranded RNA virus identified in December, 2019 in Wuhan City, China.^1^ It was initially thought to be found in pangolins, however, a rapid human-to-human transmission has occurred with a reported 15,581,009 infected individuals worldwide and 635,173 deaths (case fatality ratio 4.08%) as of 25th July, 2020.^2^

The virus attaches itself to the host cell membrane, penetrates it, releases its contents into the host cell, produces new viral proteins from the mRNA which undergo maturation. Replication occurs when the RNA enters the cell nucleus.^3^ These processes trigger a cascade of host immune responses and release of inflammatory cytokines resulting in a spectrum of clinical manifestations termed as the coronavirus disease 2019 (COVID-19).

Symptoms associated with COVID-19 are mainly respiratory as the SARS-Cov-2 has a predilection for angiotensin converting enzyme 2 (ACE2), found commonly in lung epithelial cells, heart, kidneys and gastrointestinal tract.^1^ The clinical presentations include fever, cough, difficulty breathing, chest pains, myalgia and headache.^4^ Gastrointestinal symptoms like diarrhoea and vomiting have been reported in some cases. While some infected individuals may be asymptomatic or show mild symptoms, others progress to severe disease, some of which may be life-threatening. Disease severity may be influenced by older age, comorbidities such as hypertension and diabetes, immunosuppressive conditions, male gender and ethnicity.^5^

So far, COVID-19 has shown regional variability. The hardest hit areas are South-East Asia (1,678,994 cases, 38,993 deaths [CFR = 2.32%]), Europe (3,192,041 cases, 209 930 deaths [CFR = 6.58%]) and the Americas (8,292,311 cases, 329 699 deaths [CFR = 3.98%]) .^2^ The first case of COVID-19 detected in Africa was on 14th February, 2020^6^ and it is one of the regions with the lowest statistics on the disease. As of 25th July,2020, there has been a cumulative total of 679,962 confirmed cases and 11,340 mortalities [CFR = 1.67%].^2^ There is therefore a growing interest in the disease presentation and clinical outcomes of individuals infected with COVID-19 in Africa. However, literature on this is scanty from this region. We therefore sought to share the clinical characteristics, laboratory and radiological profile and outcomes of patients with COVID-19 admitted to the University Hospital, Kwame Nkrumah University of Science and Technology (KNUST), Kumasi, Ghana.

## Methods

### Setting

The University Hospital is a 125-bed district-level facility located on the campus of KNUST, Kumasi in the Ashanti Region of Ghana. The hospital provides general and specialist services to students and staff of the University as well as private patients. It has an estimated catchment population of 200,000. Since 12th March, 2020 when the first two cases of COVID-19 were detected in Ghana,^7^ the University Hospital, KNUST has been very active in the process of case detection, testing, isolation and management of confirmed cases and contact tracing. The hospital set up a 6-bed COVID-19 Treatment Centre in June, 2020 for hospital-based management of cases. Prior to this, confirmed cases were either managed at home, admitted to an isolation centre, Kumasi South Hospital or the Komfo Anokye Teaching Hospital, based on disease severity and guidelines by the Ministry of Health, Ghana.^8^

### Sampling and Data Collection

This study captured data from a total number of 25 patients (over a period of 8 weeks) with laboratory-confirmed SARS-Cov-2 infection using real time polymerase chain reaction (RT-PCR) at the Kumasi Centre for Collaborative Research. They were all admitted in the hospital for varying duration from 1 day to 18 days and were followed up prospectively from admission till discharge. We recorded their sociodemographic data, symptoms at the time of presentation, comorbidities, blood pressure and pulse rate readings, laboratory and radiological investigations, medications given and treatment outcomes. Based on previously published guidelines, cases managed were categorized as mild, moderate, severe or critical.^3^ Laboratory reference ranges were white blood cells (WBC) 4 – 10 × 10^3^; Neutrophils 1.5 – 7.0 × 10^3^; Lymphocytes 1.0 – 3.7 × 10^3^; Hb 11.5 – 16.5 × 10^3^; platelets 140 – 400 × 10^3^ µmol/l Urea 2.5 – 8.3mmol/l; Creatinine 60 – 106µmol/l. Patients' values below the lower limit was categorised as low, those within the range was normal and those above the reference range was categorised as high.

### Data Analysis

Data was entered into Excel and descriptive analysis of parameters done then exported to STATA^®^ version 14 to determine associations among variables. Associations between mortality and demographic and clinical parameters were assessed using an exploratory multivariable logistic regression analysis. p-value of ≤0.05 was considered statistically significant.

Ethical approval was sought from the Committee on Human Research Publications and Ethics of the KNUST (CHPRE/AP/462/19).

## Results

### Demographic characteristics

Between 1st June to 27th July, 2020, 25 adults with COVID-19 were admitted to our facility. The basic demographic characteristics are shown in Table 1. In brief, there were 14 males and 11 females with no clear sex preponderance. The mean age was 59.3 (± 5.35) with a range of 21 years to 89 years. Majority (n= 15, 60.0%) were married and 44.0% (11) were retired from active service.

**Table 1:** Sociodemographic characteristics of admitted cases (N=25)

Variable	Frequency n(%)
**Age (years)**	
**21 – 30**	3 (12.0)
**31 – 40**	2 (8.0)
**41 – 50**	5 (20.0)
**51 – 60**	3 (12.0)
**>60**	12 (48.0)
***Mean Age (years) [SD]***	*59.3 (±20.63)*
**Sex**	
**Male**	14 (56.0)
**Female**	11 (44.0)
**Marital Status**	
**Married**	16 (64.0)
**Single**	4 (16.0)
**Widowed**	5 (20.0)
**Employment Status**	
**Informal Employment**	4 (16.0)
**Formal Employment**	7 (28.0)
**Retired**	11 (44.0)
**Student**	2 (8.0)
**Unemployed**	1 (4.0)

### Clinical features

In order of decreasing frequency, the commonest symptoms of hospitalized patients at presentation were difficulty breathing (17), fever (14), cough (10), confusion or altered level of consciousness (7) and anosmia (2). All of them presented with a combination of two or more of these symptoms. One of the patients reported to the hospital with mainly diarrhoea and fever.

Approximately 40.0% (n=10) of the cases were classified as severe (Table 2). Their temperatures, blood pressures (BP) and pulse rates were measured on the day of presentation, and daily until patients were discharged alive or died.

**Table 2:** Clinical Characteristics of admitted patients (N=25)

Variable	Frequency n (%)
**Hypertension**	
**Yes**	18 (72.0)
**No**	7 (28.0)
**Diabetes**	
**Yes**	9 (36.0)
**No**	16 (64.0)
**Cardiovascular Complications**	
**Yes**	6 (24.0)
**No**	19 (76.0)
**Altered consciousness**	
**Yes**	6 (24.0)
**No**	19 (76.0)
**Breathlessness**	
**Yes**	17 (68.0)
**No**	8 (32.0)
**Cough**	
**Yes**	10 (40.0)
**No**	15 (60.0)
**Fever**	
**Yes**	14 (56.0)
**No**	11 (44.0)
**Temperature**	
**Mean Temperature**	37.3 (±1.44)
**<37.5°C**	14 (56.0)
**≥37.5°C**	11 (44.0)
**Diastolic Blood Pressure (DBP)**	
**Mean DBP**	95 (±20.04)
**<90**	10 (40.0)
**≥90**	15 (60.0)
**Systolic Blood Pressure (SBP)**	
**Mean SBP**	161.5 (±27.38)
**< 140**	6 (24.0)
**≥ 140**	19 (76.0)
**Category of Cases**	
**Mild**	6 (24.0)
**Moderate**	6 (24.0)
**Severe**	10 (40.0)
**Critical**	3 (12.0)
**Outcome**	
**Discharged**	20 (80.0)
**Death**	5 (20.0)

Mean temperature was 37.3°C (±1.44) with lowest of 36°C and highest 40°C. BP ≥ 140/90mmHg was classified as high. Mean systolic blood pressure (BP) was 161.5 mmHg (± 27.38) ranging from 116mmHg and 217mmHg; mean diastolic BP 95mmHg (±20.04) ranging from 57mmHg and 160mmHg and mean pulse rate was 113.3bpm (±18.84), with the lowest being 80bpm and highest 162bpm.

### Medical comorbidities and Cardiovascular complications

Past medical history of hypertension was the commonest comorbidity comprising 18 out of the 25 (72.0%) admitted cases followed by diabetes mellitus which recorded 9 (36.0%). They had been on medications for hypertension and/or diabetes mellitus prior to admission. Of the 18 patients with hypertension, 9 (50.0%) had severe COVID-19 and three (16.7%) each were categorised as having critical, mild or moderate COVID-19. Five [27.8%] of those with hypertension had cardiovascular complications such as heart failure and ischaemic stroke. Three of the patients presented with stroke, two had left ventricular failure and one had bilateral extensive deep venous thrombosis.

**Figure 1:**
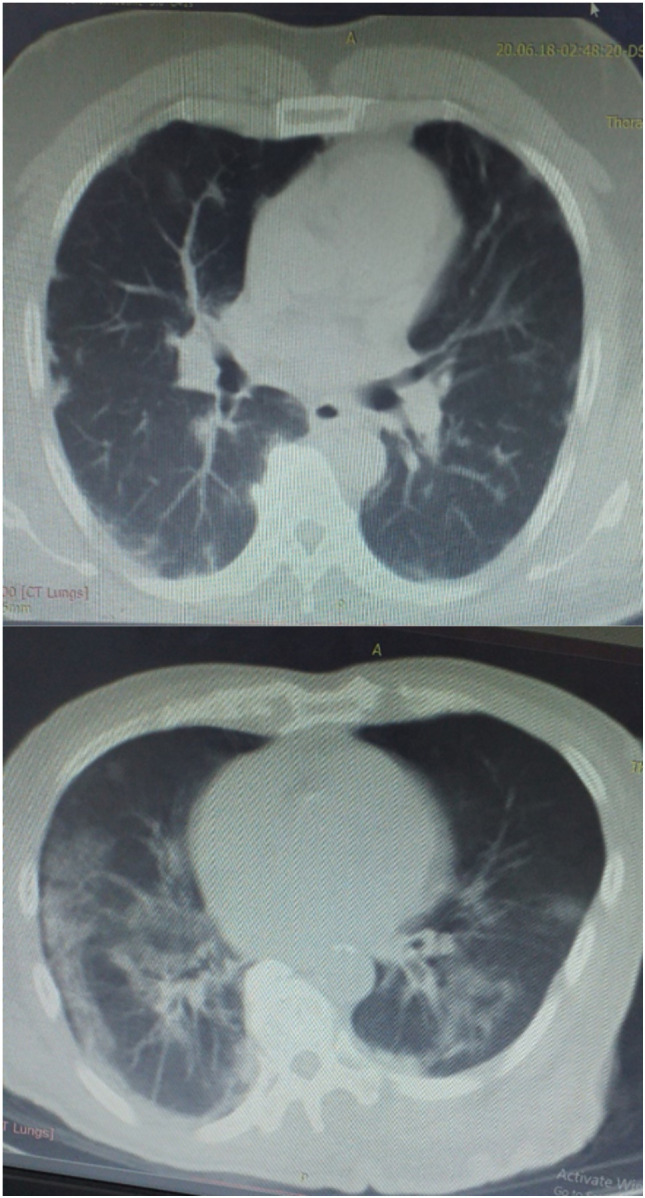
Unenhanced coronal CT images of confirmed COVID-19 cases showing the classical peripheral ground glass opacities in both lung fields

All patients with COVID-19 presenting with acute stroke died. Other comorbidities included aplastic anaemia, sickle cell disease, asthma and hepatoma with lung metastasis. Only 2 (8.0%) of the patients did not have any comorbidity. Ten (10) of the hospitalized patients had ground glass appearance and opacifications in the lungs suggestive of COVID-19 pneumonia on computed tomography (CT) scan and/or chest x-ray (Figure 1).

### Laboratory characteristics

Full blood count (FBC) data was available for 19 of the patients whilst urea and creatinine tests were done for 15. The mean absolute white blood cell (WBC) count was 10.2 × 10^3^ (± 4.96), lymphocytes was 1.99 × 10^3^ (±1.17), haemoglobin level was 12.5g/dl (± 2.64) and platelet values ranged from a minimum of 0 (patient with aplastic anaemia) to a maximum of 743 cells/ul.

Approximately 47.4% (9/19) had elevated total WBC, 42.1% (8/19) had neutrophilia and 10.5% (2/19) had neutropoenia. Lymphopoenia was observed in 10.5% (2/19) of patients and lymphocytosis was in 13.3% (2/15). Seven (36.8%) patients had haemoglobin below 11g/dl with two presenting with severe anaemia (Hbs of 7.2g/dl and 6.3g/dl). Among the different categories of patients, we observed leucocytosis and neutrophilia in one patient with critical condition, Thrombocytopenia was observed in one patient with severe condition and lymphopenia also occurred in two patients with severe condition.

The mean urea level was 7.9 mmol/l and the creatinine level was 157.15 umol/l. Of the 15 patients whose urea and creatinine tests were done, 5 (33.3%) had elevated urea only, 12 (80.0%) had elevated creatinine levels only and 3 (20%) patients categorised as severe cases had both elevated urea and creatinine, suggestive of acute kidney injury.

**Figure 2:**
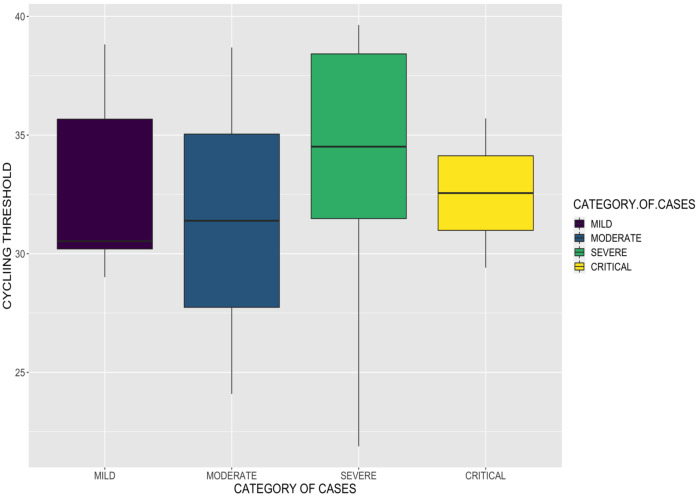
Cycle Thresholds for Category of Cases

As part of the real time polymerase chain reaction (RT-PCR) testing, we recorded the cycle thresholds (Cts) of the patients. The Cts describe the number of cycles required for the PCR fluorescent signal to exceed background level and this is inversely proportional to the starting viral load in the patient. The minimum Ct was 21.88 and the highest Ct was 39.64. Both Cts were found in severe patients. The average Ct was similar for all categories of cases (Figure 2).

### Treatment modalities

The rate of dexamethasone use was 64.0% (16) and supplemental oxygen use was 19 (76.0%). This was indicated in patients experiencing respiratory distress with oxygen saturation below 93%. Use of vitamin C and Zinc tablets was 100%, Azithromycin 96% and hydroxychloroquine use was 4%.

### Predictors of mortality

We recorded 5 (20.0%) mortalities, three of which were ‘critical’ cases and the other two, severe. The unadjusted odds ratio (95% CI) for mortality as expected increased with age with each 10-year increase in age associated with 8% (95% CI: 0 – 16%), p=0.05. Also, for each 10mmHg rise in systolic blood pressure, the odds ratio of mortality was 65% (95% CI: 1 – 169%), p=0.05.

These were exploratory analysis due to the small sample size overall and a fully adjusted analysis did not yield any independent risk factors for mortality.

## Discussion

### Symptomatology and Medications

Due to the varied presentations of COVID-19, it presents a fascinating clinical picture which challenges clinicians as well as health care systems. In Ghana, approximately 95% of individuals confirmed with COVID-19 are asymptomatic or have mild symptoms.^9^ At the University Hospital, as of 27th July, 2020, a total number of 608 individuals have been confirmed positive for COVID-19 out of which 25 (4.1%) have required hospital management. Breathlessness or difficulty in breathing was the strongest indicator for our patients to report to the hospital. This was followed by fever with 10 (40%) of them recording temperatures >37.5°C. As breathlessness is considered life-threatening, any patient experiencing it would report promptly to the hospital as compared to the vast asymptomatic majority. Reports from other centres show fever and cough as the leading symptoms with acute respiratory distress comprising 61% of hospitalized patients.^10^,^11^ The high frequency of breathlessness among our patients explains why 76% of them required supplemental oxygen.

Though the role of steroids in the management of patients with COVID-19 has been widely debated, we employed parenteral and oral dexamethasone in these patients out of which 14 were weaned off oxygen and were discharged. Dexamethasone, a glucocorticoid, has been shown to modulate inflammatory activities and ameliorates respiratory failure and death associated with COVID-19. It is reported to reduce mortality by up to 33% in patients who need mechanical ventilation and by 20% in those requiring oxygen.^12^ This is useful in our setting where there are inadequate intensive care units and ventilatory equipment. Zinc and vitamin C function as antioxidants and immunomodulatory agents which are essential in boosting a patient's innate immune response to SARS-CoV-2.^13^,^14^ All our patients, therefore, received a combination of these medications. There is evidence to support the fact that patients with COVID-19 are at risk of coagulopathy and other thromboembolic complications which increase the risk of mortality.^15^ Administering low molecular weight heparin or rivaroxaban, a factor Xa inhibitor, was to reduce this risk among 14 (58.3%) of our hospitalized patients. To date, no definitive cure has been found for COVID-19, hence, the role of these medications is mainly supportive. Besides, we could not establish any association between them and treatment outcome. Hydroxychloroquine, whose efficacy has been at the centre of global controversy, was prescribed for only one of our patients.

This was to avoid the high risk of arrhythmias associated with it, especially among patients with hypertension and cardiac problems.

Cardiac abnormalities have been found among infected patients with no previous history of cardiovascular disease.^16^ Among our patients, 72% (18) had a previous history of hypertension and 13 (52%) had episodes of tachycardia (mean pulse rate 113.3bpm) while on hospital admission.

### Laboratory Parameters

Majority of patients with COVID-19 have normal full blood count but lymphopoenia, the commonest haematological abnormality, has been detected in 28% to 63% of patients.^17^ This may be as a result of direct attack on the host's T-lymphocytes by the coronavirus. Lymphopoenia and thrombocytopaenia are strongly associated with ICU care and increased risk of COVID-19-related mortality.^17^,^18^ Lymphopoenia occurred in one of our patients who was severely ill and one patient with pancytopenia had severe thrombocytopaenia. It is yet to be determined if the patient's blood film presentation was due to COVID-19 or purely a case of aplastic anaemia. Leucocytosis with elevated neutrophils, which were also observed in five of our patients, may be attributed to the hypothesis that these cells could be recruited early to sites of infection where they kill pathogens by oxidative burst and phagocytosis.^19^ Generally, neutrophils are spared by SARS-CoV-2 in the initial phase of the disease.^19^

Abnormal levels of urea and creatinine among our patients are similar to studies from other COVID-19 treatment sites. Angiotensin-converting enzyme 2 (ACE-2) receptors expression in human kidneys, bladder and testis reveals a potential route of coronavirus-mediated renal dysfunction, which may cause death in infected patients.^20^ Approximately 70.8% (17) of our patients had elevated urea, creatinine or both, supporting the high incidence of acute kidney injury among COVID-19 patients reported elsewhere.^21^ This may be related to the severity of the disease.

Apart from the clinical presentations, we sought to assess the disease severity based on the cycle threshold (Ct) or viral load of patients recruited into this study. However, we could not establish this since the Cts recorded were similar for all categories of cases. In a previous study, viral load was undetectable in early stage of some infected patients.^22^ Since the Cts of our patients were assessed once, at the time of COVID-19 diagnosis, future studies should include evaluating Cts at different time points. Our relatively small sample size may also have contributed to this, as well as the varying haematological and biochemical parameters which did not have any statistically significant association with COVID-19 severity or outcomes. Thus, we recommend further laboratory studies of a bigger cohort of patients with COVID-19.

### Risk Factors for Mortality

Mortality is relatively high among hospitalized patients as 5 out of 25 patients succumbed to the disease (Case Fatality Ratio [CFR] 20.0%). In the United Kingdom, among hospitalized patients, the CFR is between 26 to 37%^12^, 4.3 to 13% in China^23^ and 7.2% in Italy.^12^ The mortalities in our study involved three males and two females who were above 60 years old and all of them had hypertension and/or diabetes as a comorbidity. Although we cannot make generalizations due to our small sample size and the lack of statistical significance, it has been shown that predictors of COVID-19 deaths include male gender, older age and underlying medical conditions.^23^,^24^ In an exploratory analysis, there were suggestions that increasing age and higher systolic blood pressure at presentation may be associated with risk of mortality. However, due to the limited sample size of our study, neither age nor blood pressure was found to be independently associated with risk of mortality. A larger study may be required to characterize the risk factors associated with mortality among patients hospitalized with COVID-19 in Ghana. Each 10-year rise in age was associated with 8% (95% CI: 0 – 16%) higher odds of death while each 10 mmHg rise in systolic blood pressure was associated with 65% higher odds of mortality. However, both covariates failed to achieve statistical significance in adjusted analysis. These observations underscore the importance of rigorous management of cardiovascular comorbidities among hospitalized COVID-19 patients. Indeed, the disruptions to healthcare delivery engendered by this outbreak have been associated with a rise in stroke admissions notable recurrent strokes and a trend towards increased mortality from stroke.^24^ The University Hospital, KNUST is a district-level hospital whose COVID-19 treatment centre was designated for management of mild-to-moderate cases. Lacking an intensive care unit and other essential specialists, severe and critically ill patients were supposed to be transferred to the only teaching hospital in the region. However, Ghana has a perennial problem of ‘no bed syndrome’^25^ which has been heightened during the COVID-19 pandemic, making it difficult for us to step-up care for some of our patients who needed it. In this study, we did not explore how much this contributed to the deaths of our patients who had severe-to-critical COVID-19.

### Limitations and Strengths

We relied solely on data of confirmed cases of COVID-19 at the hospital. Working in a resource-limited setting, we were constrained by a lack of resources to conduct the same laboratory and radiological investigations for all the patients. Some markers of disease severity such as d-dimer and lactate dehydrogenase were not employed. Decisions to undertake some tests were mainly based on patients' clinical picture. This, notwithstanding, this paper is a significant step in providing information on the clinical and diagnostic profile of hospitalized patients with COVID-19 in Ghana.

## Conclusion

We have demonstrated the contrasting but intriguing clinical and laboratory presentation of COVID-19 among a small sample of confirmed cases hospitalized in Ghana. Cardiovascular complications, haematological and renal abnormalities are common. Although there was no independent predictor of treatment outcomes, risk factors that require critical attention include older age and comorbidities such as hypertension. We encourage multi-centre bigger studies on COVID-19, especially in sub-Saharan Africa.
